# Diabetes and Alzheimer Disease, Two Overlapping Pathologies with the Same Background: Oxidative Stress

**DOI:** 10.1155/2015/985845

**Published:** 2015-02-26

**Authors:** Sergio Rosales-Corral, Dun-Xian Tan, Lucien Manchester, Russel J. Reiter

**Affiliations:** ^1^Centro de Investigación Biomédica de Occidente, Instituto Mexicano del Seguro Social, Sierra Mojada 800, Colonia Independencia, 44340 Guadalajara, JAL, Mexico; ^2^Department of Structural Biology, The University of Texas Health Science Center at San Antonio, 7703 Floyd Curl Drive, San Antonio, TX 78229-3900, USA

## Abstract

There are several oxidative stress-related pathways interconnecting Alzheimer's disease and type II diabetes, two public health problems worldwide. Coincidences are so compelling that it is attractive to speculate they are the same disorder. However, some pathological mechanisms as observed in diabetes are not necessarily the same mechanisms related to Alzheimer's or the only ones related to Alzheimer's pathology. Oxidative stress is inherent to Alzheimer's and feeds a vicious cycle with other key pathological features, such as inflammation and Ca^2+^ dysregulation. Alzheimer's pathology by itself may lead to insulin resistance in brain, insulin resistance being an intervening variable in the neurodegenerative disorder. Hyperglycemia and insulin resistance from diabetes, overlapping with the Alzheimer's pathology, aggravate the progression of the neurodegenerative processes, indeed. But the same pathophysiological background is behind the consequences, oxidative stress. We emphasize oxidative stress and its detrimental role in some key regulatory enzymes.

## 1. Introduction

Three hundred forty-seven million people worldwide have diabetes [1], and forty-four million people live with dementia [2].

An inadequate glucose metabolism in the brain resulting from insulin resistance, the reduced ability of insulin to stimulate glucose utilization, is at the center of new therapeutic avenues to treat the most common cause of dementia worldwide, that is, Alzheimer's disease (AD). A recent study showed that over a maximum 11 years of follow-up, diabetic patients experienced a higher incidence of AD than nondiabetic subjects [3]. Moreover, it is postulated that AD represents a neuroendocrine disorder that resembles a unique form of type 2 diabetes mellitus (T2D) accompanied by neurodegeneration, which is sometimes considered type III diabetes [4]. Derived from this hypothesis, some agents that improve insulin sensitivity and reduce hyperinsulinemia have been proposed to aid cognitive functioning for patients with T2D or AD.

Oxidative stress (OxS) plays a major role in T2D [5]. At the same time, all the proposed mechanisms to explain AD pathology have a common factor: OxS. Additionally, there is a well-known connection between the main pathological features of AD, such as amyloid-beta (A*β*) protein and hyperphosphorylated tau, with glucose metabolic intermediates, insulin receptors, and insulin transporters, all in the same context: OxS. In fact, the chemical depletion of insulin and insulin-like growth factor (IGF) signaling mechanisms plus oxidative injury seem to be sufficient to cause AD-type neurodegeneration, as demonstrated* in vivo* using a model of intracerebral streptozotocin administration [6].

**Figure 1:**
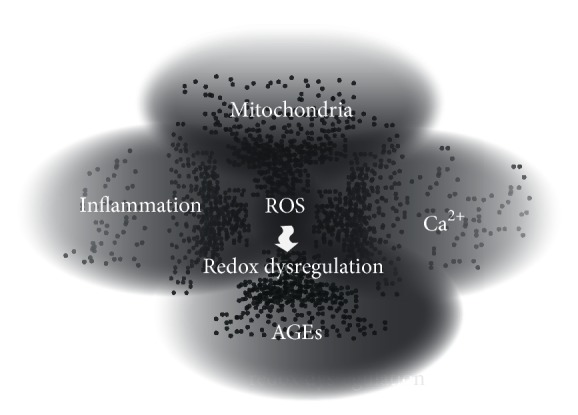
Hyperglycemia and insulin resistance induce free radicals which are responsible for tissue damage. The main sources for free radicals are mitochondrial dysfunction, cytosolic free Ca^2+^, the vicious cycle between inflammation and OxS, and the activity of advance glycation end products, which promote the innate immune response through their receptors. These conditions produce a significant pool of free radicals, sufficient to cause OxS in the brain. Excessive amounts of ROS/RNS break the delicate regulation of key signaling and effector proteins required to maintain the homeostasis in the brain. ROS: reactive oxygen species; AGEs: advanced glycation end products.

Hyperglycemia and insulin resistance likely have an impact on OxS pathways and neuroinflammatory signals in the brain, thereby connecting diabetes to neurodegeneration. Four pathological-free radical sources in the brain have been described which also feed the OxS in diabetes: mitochondrial dysfunction, inflammation, advanced glycation end products (AGEs), and high cytosolic ionic-calcium levels. A particular issue with regard to OxS is the redox-dysregulation, a relevant condition which demands more attention (Figure 1).

It is the purpose of this review on the basis of these observations, to analyze the interrelationships between OxS and redox-regulation in T2D-related pathways and AD pathology.

## 2. Consuming the Antioxidant Substrates

It has been hypothesized that OxS could be the common pathogenic factor leading to insulin resistance and *β*-cell dysfunction in T2D [7], as well as the vascular events [8] associated with this global epidemic disease [9]. Reactive oxygen species (ROS) derived from an uncontrolled T2D come mainly from the polyol pathway flux, which consumes the equivalent reducers, essential cofactors for redox systems responsible for scavenging free radicals.

The first enzyme in the polyol pathway, aldose reductase (AR), reduces glucose to sorbitol, which is then transformed to fructose by sorbitol dehydrogenase (SDH) [10]. AR has low substrate affinity for glucose, such that high concentrations of glucose are needed. Hyperglycemia pushes the polyol pathway and AR consumes NADPH to transform glucose into sorbitol. NADPH, nonetheless, is essential for reducing glutathione disulfide (GSSG, the oxidized glutathione) to glutathione (GSH) in a critical reaction to control free radical levels within cells; this is carried out by the enzyme glutathione reductase. The impaired cognitive function associated with hyperglycemia may be corrected by inhibiting the polyol pathway and normalizing sorbitol and taurine in the brain, even without correcting the extracellular hyperglycemia, as demonstrated experimentally* in vivo* [11].

GSH depletion reduces the capability of cells to remove ROS, making the oxidative processes irreversible [12]. A negative linear correlation between GSSG levels and cognitive status in AD patients has been found [13, 14]. This is such an important correlation that GSH has been considered useful as a biomarker for AD progression [15], and a measurable GSH deficiency is found in T2D [16], as explained below.

GSH is synthesized from glutamate, cysteine, and glycine and diabetic patients have been shown deficient in both cysteine and glycine [17]. It is suggested that such a deficiency is due to a combination of impaired protein turnover and dietary deficiency [17]. Patients with uncontrolled T2D have 74% lower erythrocyte-reduced glutathione concentrations than nondiabetic control subjects, and a higher concentration of erythrocyte-oxidized GSSG. Once the GSH synthesis is restored by dietary supplementation with cysteine and glycine, reactive oxygen metabolites and lipid peroxides may be significantly lowered [18].

GSH demand, without replenishment, leads to GSH depletion. The restoration of GSH levels depends on the cysteine into cells via an amino acid antiporter system Xc^−1^ to import extracellular l-cystine and export intracellular l-glutamate across the cellular plasma membrane. It is worth remembering that glutamate excitotoxicity is a key protagonist in AD pathogeny [19–21], and the source of glutamate comes precisely from this cystine-glutamate antiporter. In fact, microglia may release glutamate by system Xc^−1^ altering glutamate homeostasis, as shown both in oligodendrocytes and in isolated optic nerve fibers [22]. The transportation of cystine into cells, rate limiting for glutathione synthesis and catalyzed by the glutamate cysteine ligase (GCL), as well as the antiporter system Xc^−1^ itself, is significantly affected in T2D [23–25], particularly during ketoacidosis [26].

**Figure 2:**
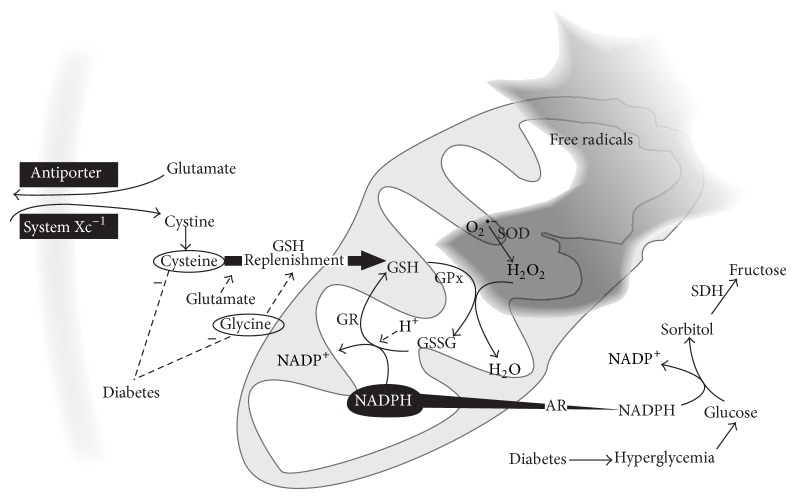
Mitochondria are the main source of free radicals in neurodegenerative diseases, which is particularly true in the Alzheimer brain. The tripeptide GSH is formed in the cytosol from cysteine, glutamate, and glycine as substrates (glutamate reacts with cysteine in the presence of *γ*-glutamylcysteine ligase to produce *γ*-glutamylcysteine, which in turn reacts in a second step catalyzed by the enzyme GSH synthetase with glycine, to produce GSH). From the cytosol, GSH is distributed to the nucleus, endoplasmic reticulum (ER), and mitochondria. GSH is a key, abundant antioxidant system to control free radical overproduction in the central nervous system. As long as GSH can be replenished, a reducing intracellular environment prevails, depending on the amount of substrates for its synthesis and the proper functioning of the antiporter system, Xc^−1^. In T2D, the polyol pathway consumes NADPH to transform glucose into sorbitol, affecting the GSH system. Conversely, the two important substrates for GSH replenishment, cysteine and glycine, are reportedly diminished in T2D. SOD: superoxide dismutase; GR: glutathione reductase; GPx: glutathione peroxidase; GSH: *γ*-l-glutamyl-l-cysteinyl-glycine; GSSG: oxidized glutathione; NADPH: reduced form of NADP^+^ nicotinamide adenine dinucleotide phosphate; AR: aldose reductase; SDH: sorbitol dehydrogenase.

Another important source of NADPH for antioxidant systems comes from the main energy-transducing metabolic systems. A major source of NADPH is the glucose-6-phosphate dehydrogenase (G6PD), which converts glucose to ribose-5-phosphate. During hyperglycemia, G6PD activity decreases significantly; this correlates with low levels of NADPH and reduced glutathione (Figure 2), as observed in kidney cells. The inactivation of G6PD is strongly correlated with phosphorylation of its serine residues by the protein kinase A (PKA) [27]. This cAMP-dependent kinase phosphorylates serine/threonine residues, it is dependent of hyperglycemia, and it is also related to phosphorylation of tau in the AD brain [27, 28]. It is worth mentioning that PKA is a ubiquitous enzyme and its dependence on hyperglycemia is a relevant condition to memory processes in the brain. This is particularly relevant to the AD brain, since PKA is involved in the development of long-term potentiation via cAMP response element-binding protein (CREB) phosphorylation. In fact, cAMP/PKA/CREB enhancers, such as rolipram and forskolin (PKA activators), have been proposed as useful to memory impairment [29] and also for AD treatment since one of the main protagonists in this neurodegenerative disorder, amyloid-beta (A*β*), also inhibits the PKA/CREB pathways and long-term potentiation [30]. Additionally, PKA modulates NMDA receptors as well, and this is relevant to excitotoxic neurotransmission and calcium homeostasis, since PKA activates calcium release channels [31, 32].

Another important metabolic source of NADPH to replenish antioxidant systems comes from the NADP^+^-dependent isocitrate dehydrogenase (IDH). However, IDH is particularly susceptible to fragmentation and carbonylation when exposed to reducing sugars, such as glucose, glucose-6-phosphate, and fructose [33] (Figure 2). Thus, glycation-induced inactivation of NADP^+^-dependent IDH during T2D progression ultimately leads to failure to produce NADPH, as it should occur during the oxidation of isocitrate to the intermediate oxalosuccinate in the tricarboxylic acid cycle. The reduced amount of NADPH correlates with significant increases in ROS generation, DNA fragmentation, lipid peroxidation, and concurrent mitochondrial damage with a significant reduction in ATP levels [34].

Mitochondrial ATP production in T2D is significantly diminished indeed. An evaluation of mitochondrial phosphorylation using proton 1(H) magnetic resonance spectroscopy in muscle of lean, prediabetic insulin resistant subjects, showed that mitochondrial phosphorylation and thus ATP production may be only 30% of controls [35].

A causal relationship between the mitochondrial overproduction of free radicals plus lipid peroxidation and hyperglycemia was shown* in vitro* with cultured bovine aortic endothelial cells. ROS overproduction exhibited a positive correlation with intracellular AGEs generation, an effect prevented by using antioxidants [36]. Years later in similar experiments, after normalizing mitochondrial superoxide production, AGEs overproduction, protein kinase C (PKC) activation, increased glucose flux through the aldose reductase pathway, and NF-*κ*B activation, were blocked [37].

Another important source of free radicals in AD comes from their rapid release during the respiratory burst, particularly from activated microglia. Again, the AR enzyme from the exacerbated polyol pathway in T2D could be involved, since the transformation of sorbitol to fructose by the enzyme sorbitol dehydrogenase reduces NAD^+^ to NADH. NADH in turn is essential as a reducing equivalent for the NADPH oxidase, which orchestrates the respiratory burst. Thus, it is feasible to speculate that the polyol pathway may feed the NADPH-oxidase [38]. All of these signs of OxS are key pathogenic events during AD progression [21]; they are actually intrinsic to AD pathology.

**Figure 3:**
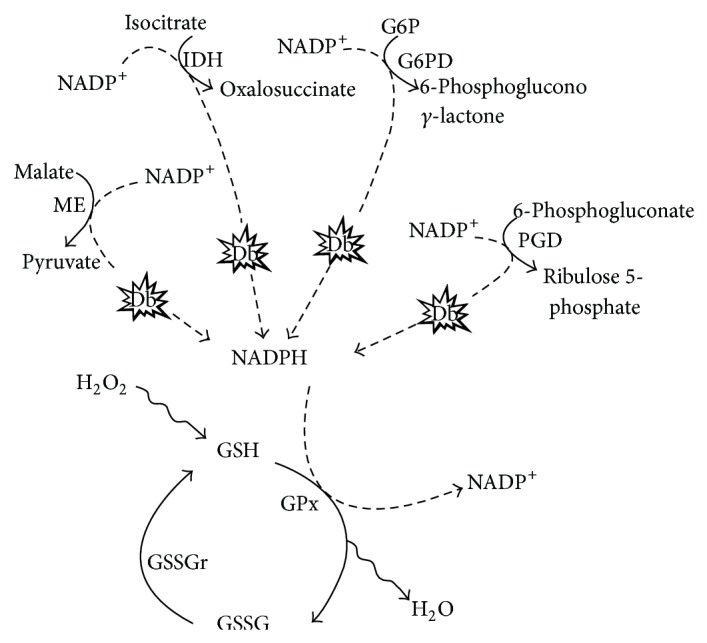
T2D and metabolic NADPH sources related to GSH functioning. 6-Phosphogluconate dehydrogenase is also reduced in experimentally induced T2D. IDH: isocitrate dehydrogenase; G6PD: glucose-6-phosphate dehydrogenase; PGD: phosphogluconate dehydrogenase; ME: malic enzyme; Db: T2D; GPx: glutathione peroxidase; GSSGr: glutathione reductase.

Another source of NADPH is the malic enzyme (ME, L-malate: NADP oxidoreductase (decarboxylating)) which catalyzes the reversible formation of pyruvate, CO_2_, and NADPH from malate and NADP. Malic enzyme has been studied mostly in liver where it supplies reducing equivalents from NADPH for fatty acid biosynthesis. In the brain, cytosolic ME is located in oligodendrocytes where it might deliver NADPH for myelin lipid biosynthesis, whereas in astrocytes it is presumed to participate in the catabolism of excessive Krebs cycle intermediates [39]. Interestingly, the mitochondrial isoform of ME is abundant in neurons, which allows the speculation that it serves as a GSH regeneration system [40–42]. Finally, ME levels are reportedly diminished in T2D, probably linked to a depressed lipogenesis [43] (Figure 3). Thus, after this brief analysis of NADPH sources in brain, it is clear that the high demand of NADPH in T2D, plus a failure to replenish the GSH system, favor OxS, and the progression of AD and/or T2D pathologies (Figure 3).

GSH depletion also induces apoptosis on hippocampal neurons by perturbing calcium (Ca^2+^) homeostasis, as demonstrated in aged mice [31]. Apoptosis and alterations of ionic calcium are intrinsically linked to AD [21].

## 3. Thioredoxin (Trx)

Working at the expense of NADPH as well, Trx with its active sequence –Cys-Gly-Pro-Cys- is essential to reduce oxidized proteins by cysteine thioldisulfide exchange. It is abundant in brain, particularly in those regions with high energy demands [44]. Trx reduces peroxidases, methionine sulfoxide reductases, sulfate reductases, or the ribonucleotide reductase, acting as an electron donor [Trx(SH)_2_ + ROOH → TrxS_2_ + ROH + H_2_O]. NADPH further reduces the oxidized Trx. This small oxidoreductase enzyme is essential to maintain the redox status [12].

Activity and availability of Trx, however, can be controlled by alkylating agents such as 4-hydroxynonenal (HNE) which forms thiol adducts, as observed in AD pathology [45]. In T2D, the OxS mediator thioredoxin-interacting protein (TxNIP) plays a key role in Trx inactivation, linking OxS to glucotoxicity. During that process, TxNIP triggers the inflammasome activity as well.

The TxNIP gene appears elevated in insulin resistance/T2D and it is upregulated by glucose [46]. It is implicated as a disease-driver in both pancreatic islets by mediating glucose-induced cell death [47]. In brain, TxNIP is induced in neurons after OxS, chronic hyperglycemic stress, endoplasmic reticulum stress, or ischemia and causes cells to undergo apoptosis [48, 49].

Hyperglycemic signals activate TxNIP through the transcription factor carbohydrate response element-binding protein (ChREBP), which translocates to the nucleus to bind the carbohydrate responsive element (ChoRE) [50] located in the promoter regions of both glycolytic (L-PK) and lipogenic genes (ACC and FAS). Under glucose stimulation, ChREBP uses a tandem ChoRE and CCAAT motifs with the collaboration of the nuclear factor Y (NF-Y) to regulate TxNIP [51].

Impaired insulin signaling in AD brain seems to be related to TxNIP as well. This major intracellular regulator of inflammatory activation and redox stress has been found early overexpressed very early in the brain of the 5XFAD Alzheimer mice model. Thus, TxNIP is also considered a key factor in the insulin resistance and can be induced in astrocytes, endothelial, and neuronal cells* in vitro*, by adding A*β* to the medium [52].

## 4. Amplifying the Inflammatory Response

The link between the nucleotide-binding domain, leucine-rich-repeat-containing family, pyrin domain-containing 3 (NLRP3) inflammasome assembly, and TxNIP, readily induced by hyperglycemia, seems to be a transmembrane sensor protein with both kinase and ribonuclease activity known as the inositol-requiring protein-1 alpha (IRE1*α*). IRE1*α* is a highly conserved ER sensor and plays a key role during the unfolded protein response in the brain [53]. It is related to Ca^2+^ homeostasis and cell survival during ER stress [54], this latter process is a relevant phenomenon in the pathogenesis of AD [49, 54–57]. Thus, under ER stress IRE1*α* becomes active and it is required to promote TxNIP [49]. TxNIP activates the NLRP3 inflammasome and promotes programmed cell death under unremediated ER stress.

Via stimulation of G protein-coupled calcium sensing receptors, extracellular Ca^2+^ may amplify the inflammatory response activating the NLRP3 inflammasome assembly, as shown in monocytes and macrophages [58]. The process occurs via the inositol/Ca^2+^ pathway leading to the release of high levels of interleukin 1*β* and other proinflammatory cytokines, such as IL-1*α*, IL-6, and TNF. A kinetic curve of proinflammatory cytokines during A*β*-induced OxS in brain shows the same pattern of cytokines as a function of time [59].

TLR (toll-like receptor) is the priming step to activate NF-*κ*B transcription factor, which initiates the NLRP3 multiprotein complex. Stimulation by extracellular ATP may be the second signal to culminate in the complete organization of these large cytosolic complexes of NOD-like receptors, adaptor protein (which is an apoptosis-associated speck-like protein containing a CARD), and caspase-1. In AD brain, the appearance of extracellular ATP, which assumes a role as a damage-associated molecular pattern molecule (DAMP), is linked to bioenergetic dysfunction [60]. NLRP3 culminates in the activation of caspase-1 and releases IL-1*β* and IL-18, proinflammatory cytokines [58, 61, 62].

NLRP3 induces metabolic inflammation and, importantly, insulin resistance. By deactivating it as happens in obese diabetic patients with a significant loss of weight, insulin resistance diminishes [62]. Furthermore, the elimination of Nlrp3 expression may prevent the activation of caspase-1 as observed linked to obesity and activation of IL-1*β* and IL-18. Free fatty acids in obese subjects also may induce DAMP, which are linked to the amplification of the innate immune response as occurs in T2D. Free fatty acids also induce insulin-resistance [63] and extracellular ATP [64].

In AD, ATP molecules are also delivered to the extracellular space assuming a new role as DAMP. Thus, extracellular ATP takes part in the innate immune receptor surveillance, as occurs with the amyloid-induced inflammasome [65]. In this manner, extracellular ATP molecules may elicit Ca^2+^ waves (ATP-dependent glial-transmission) and DAMP-mediated activation of microglia, which in turn activate the phagocytic NADPH-oxidase [66]. This multimeric membrane-bound enzyme complex links redox control to the neuroinflammatory signaling pathways [67]. Similar actions are observed for the T2D-induced inflammasome.

Hyperglycemia in T2D produces a nonenzymatic glycation and oxidation of proteins and lipids which are known as AGEs. In the AD brain, both AGE and A*β* are abundant and both utilize scavenger receptors (SR) and the receptor for AGE (RAGE). SR and RAGE may define microglia activity [68], but they are also expressed in astrocytes. Also neurons may express RAGE [69]. This calls the attention to the fact that RAGE appears overexpressed in AD brain [70], while RAGE's activation in vessels may result in amplification and perpetuation of a loop for OxS and dysregulation of proinflammatory cytokines [71].

The key target of these receptors is NF-*κ*B, the transcription factor where the neuroinflammatory pathways converge. RAGE and NF-*κ*B are upregulated during hyperglycemia, as observed in the hippocampus of rat brain [58, 69, 72, 73]. Upregulation of RAGE and NF-*κ*B is accompanied by overactivation of inflammatory factors such as TNF-*α*, IL-1*β*, IL-2, and IL-6.

It is important to remember that insulin, acting on its own insulin receptor kinase (IRK), may directly stimulate the NADPH-oxidase pathway, generating an H_2_O_2_ burst [74, 75]. Thus, insulin itself may feed OxS, contributing to the vicious cycle of OxS-inflammation.

## 5. Calcium

The disturbed Ca^2+^ homeostasis is relevant to AD. Ca^2+^ is related to acetylcholine expression and its metabolism as well as the activity of its receptor [76]. Ca^2+^/calmodulin regulate protein phosphorylation and there are specific calcium-dependent signal transduction pathways in AD neurodegeneration implicating key protein effectors, such as calmodulin-dependent protein kinases (CaMKs), mitogen-activated protein kinases (MAPK), and CREB. Importantly, Ca^2+^ waves are a kind of communication between cells in the brain during neuroinflammation which regulate glial cells [21, 65]. A*β*, as part of AD pathology, also enhances the acetylcholinesterase (AchE) activity and induces significant elevations of intracellular Ca^2+^ by increasing calcium entry through L-type voltage-dependent calcium channels [77–79]. AchE releasing is a Ca^2+^-dependent phenomenon [77, 80].

Ca^2+^ in AD is related to multiple effects: (1) amyloidogenic processing of the amyloid precursor protein (APP) seems to be a Ca^2+^-dependent process; (2) A*β* facilitates Ca^2+^ uptake using a variety of calcium channels; (3) conversely, Ca^2+^ accelerates A*β* aggregation; (4) the massive entrance of extracellular Ca^2+^ into cells causes excessive accumulation of Ca^2+^ within the endoplasmic reticulum and mitochondria; (5) dysregulated intracellular Ca^2+^ activates a number of enzymes including CaM, which in turn activates CaM-dependent kinases responsible for tau phosphorylation; (6) cytosolic phospholipase-A2 (PLA2) is also a Ca^2+^-dependent enzyme which, when activated, causes arachidonic acid release and the subsequent activation of the neuroinflammatory pathway via COX-2. Additionally, the massive influx of Ca^2+^ promotes proteolytic calpains and neuronal death through the Ca/CaM dependent kinase II and a divergent number of enzymes (reviewed in [21]).

It is feasible to reproduce Ca^2+^ mobilization linked to glutamate exocytosis in T2D [81]. The proposed mechanism is that T2D enhances a K^+^- or 4-AP-evoked Ca^2+^-dependent glutamate release by increasing the concentration of free cytosolic Ca^2+^ via stimulation of Ca^2+^ entry through both P- and N-type Ca^2+^ channels [81]. In T2D, in addition to the mentioned Ca^2+^ influx into cytosol through P- and N-type Ca^2+^ channels, hyperglycemia also may cause hyperglycosylation of Ca^2+^ channel CaV3.2 and/or membrane lipids that affect channel function causing an increase in current density [82].

The increased cytosolic concentration of Ca^2+^ is related to mitochondrial damage and overproduction of free radicals. Ca^2+^ uptake into mitochondria induces neuritic abnormalities in a dose- and time-dependent manner or the opening of the mitochondrial permeability transition pore coupled to inhibition of respiratory complexes [83]. Another means by which OxS-induced Ca^2+^ may be relevant to both pathologies, AD and T2D, is by amplifying the inflammatory response, a ROS-induced phenomenon where extracellular Ca^2+^ may have a key role, as seen before.

## 6. Redox Regulation of Key Enzymes

Proinflammatory pathways are redox-regulated processes aided by redox sensors. These thiol-based redox sensors convey information about localized changes in redox potential induced by physiologic or pathologic situations. However, the persistence of OxS keeps progressive pressure on the effective reduction potential of these sensors. The NADPH-dependent GSH/GSSG and the thioredoxin system work as redox sensors, but there are thousands of peptidyl-Cys residues that are redox-sensitive and may work as redox-regulators [84].

The insulin receptors themselves are tyrosine kinases with critical thiol groups which are necessary for the beta-subunit autophosphorylating activity, and they are redox-regulated enzymes. A reduced expression of insulin/IGF receptors in AD, as well as their receptors and their substrates, has been observed [4, 85]. Such a deficiency may be attributed to a progressive loss of insulin/IGF responsive neurons, or to impaired insulin/IGF ligand-receptor binding, due to pathological alterations in membrane lipid composition [86]. However, in a study of brain insulin receptors, following the intracerebroventricular injection of streptozotocin, insulin receptor levels were shown to be diminished. The low IRK levels were inversely correlated with the degree of OxS, indicated by malondialdehyde (MDA) and GSH levels. By reducing OxS with melatonin, a powerful antioxidant and free radical scavenger, it was possible to reverse the IRK diminishing [87]. Also, an insulin-independent “basal” insulin receptor kinase activity has been described and is strongly enhanced by H_2_O_2_ or by an oxidative shift in the redox status [88].

This redox-regulatory effect on IRK seems to respond to a positive feedback; this is an autoregulatory mechanism, since IRK is capable of inducing H_2_O_2_ by activating NADPH-oxidase. However, in opposition to low, regulatory H_2_O_2_ levels, OxS inhibits insulin signaling with the consequent inactivation of the Akt/PKB signaling pathway, plus the impairment of GLUT4 translocation, all resulting in insulin resistance [89, 90]. By employing the same redox-regulatory mechanism, H_2_O_2_ may also inhibit key components of the IP3/DAG pathway (Inositol 1,4,5-trisphosphate/diacylglycerol), such as the protein tyrosine phosphatase (PTP1B) and the protein phosphatase and tensin homolog (PTEN) [91].

PTP is a negative regulator of insulin and leptin signal transduction, considered a novel target for T2D treatment [92]. IRK phosphorylates the insulin receptor substrate (IRS), which displays binding sites for numerous signaling partners, thereafter a complex set of reactions, orchestrated by phosphatidylinositol-3-kinase (PI3K) connect to the Akt/PKB signaling pathway and PKC. PTEN catalyzes the reverse reaction of PI3K regulating the phosphorylation state of phosphatidylinositol (3,4,5)-trisphosphate (PIP3), a membrane lipid second messenger; PIP3 is therefore a key mediator of the AKT/PKB pathway. These intermediates in turn connect insulin to vital processes such as, mitogenic and stress pathways (IRK → Ras → MAPK → ERK), and metabolic pathways (IRK → PI3K → PDK1 → AKT).

PTP dephosphorylates and inactivates the IRK, reversing the adapter function of the IRS. In fact, the specific PTP-1*β* has become a prime candidate for therapeutic intervention in diabetes and obesity [74]. These phosphatases have highly conserved cysteine residues within the active site domain, with a low-pKa (4.7 to 5.4), in such a manner that different reactive oxygen species, including H_2_O_2_, oxidize and inactivate the PTP. However, thiol donors, such as GSH, glutaredoxin (Grx), and Trx may rescue thiol groups from irreversible oxidation; via this means the enzyme inactivation can be reversed. Thus, PTP are redox-regulated enzymes [93, 94].

Interestingly, insulin by itself may generate a burst of intracellular H_2_O_2_ as a result of its stimulation of the NADPH-oxidase pathway. H_2_O_2_, in turn, induces to a significant reduction in overall PTP activity and enhances the abovementioned insulin cascade. The effect has been shown to be reversed* in vitro* by suppressing H_2_O_2_ with the addition of catalase (2H_2_O_2_→ 2H_2_O + O_2_) [75].

Beyond tuning and redox regulation, it is important to highlight that as long as the oxidative pressure increases, the effective redox reduction potential of the redox sensors diminishes. This shifts cell signaling toward proinflammatory pathways, leading to inflammation, apoptosis, and more OxS. Ultimately, a vicious cycle between OxS and neuroinflammation develops [12]. One of the main prooxidant pathways related to hyperglycemia is the PKC signaling pathway; however, the role for this redox sensitive serine/threonine kinase is controversial in AD neurodegeneration.

**Figure 4:**
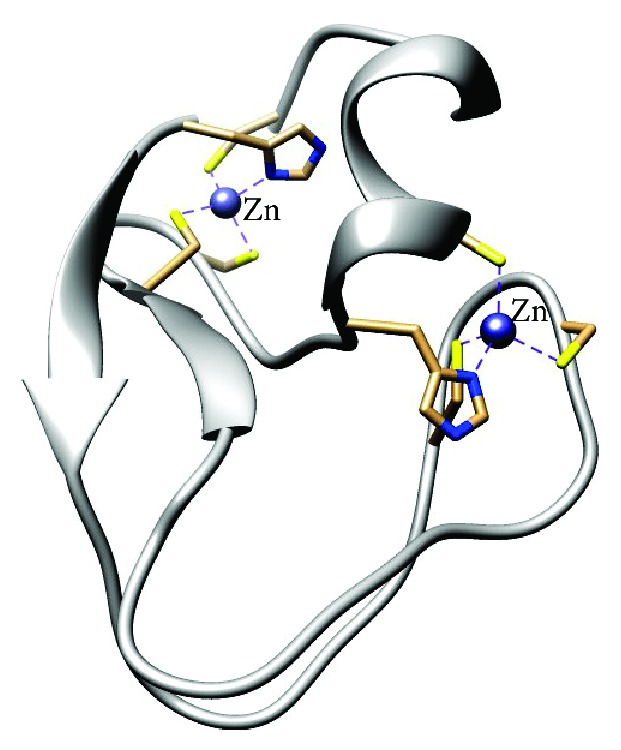
Coordinated Zn bindings with cysteine residues in the catalytic and the regulatory domain of PKC. This figure was generated from pdb entry 1PTQ [95] using the UCSF Chimera package [136].

PKC contains six conserved cysteine and two conserved histidine residues tetrahedrally coordinated by two Zn^2+^ ions into a composite zinc finger [95]. There are variations among the different PKC isozymes related to their responses to OxS; some become active and others became inactive in the same tissue and under a similar context. However, all PKC proteins dissociate from Zn^2+^ after H_2_O_2_ treatment [96]. Furthermore, cysteine-rich regions present in the regulatory domain are the sites of phorbol ester/diacylglycerol binding [97] (Figure 4).

PKC can be S-glutathiolated and inactivated during OxS in brain. In an oxidizing microenvironment, disulphide formation due to an oxidative attack dissociates Zn^2+^, which becomes uncoupled [98]. Zn^2+^ is required to maintain the redox homeostasis, and it must be rapidly buffered to reestablish its levels in neurons. By occurring on oxidation within the kinase domain the inactivation of PKC may be reversible, as an adaptive mechanism in response to stress. However, a severe oxidation with disulfide bond formation can be irreversible, leading to protein degradation and apoptosis [99].

PKC has cysteine thiols susceptible to redox-regulation in both the regulatory domains as in the catalytic domain [100] (Figure 4). Examined in tumorigenesis, the oxidation of these thiols may cause an opposite response: oxidation of thiols on the regulatory domain and stimulation of PKC, leading to tumor promotion and cell growth. In contrast, oxidation of thiols in the catalytic domain inhibits PKC activity, interfering with tumor promoters.

In PC12 cells, OxS causes direct redox activation of PKC-*ε*, which in turn leads to a rapid and sustained activation of ERK, necessary and sufficient for neurite outgrowth in these cells [101]. ERK activation in diabetes is linked to diabetic microvascular disease related to the activation of the transcription factor hypoxia inducible factor-1 (HIF-1) [102].

There are many isoforms of PKC and every isoform has some particular mechanisms of regulation and its specific downstream signaling mechanisms [74, 95, 96, 99, 101]. There are classical PKCs (*α*, *β*I, *β*II, and *γ*), novel PKCs (*ε*, *δ*, *η*, and *θ*) which depend on DAG alone without the participation of Ca^2+^, and also atypical PKCs (*ζ* and *ι*). The specific roles and the mechanisms of activation or deactivation for each isoform are not completely clarified. This explains, perhaps, some discrepancies about how PKC become involved in certain pathologies.

Hyperglycemia activates different PKC isoforms through OxS or by employing the DAG/IP3 pathway. Additionally, insulin induces superoxide anion via direct stimulation of the NADPH-oxidase, as mentioned above. Superoxide anion is dismutated to H_2_O_2_ via SOD and it has been demonstrated that H_2_O_2_ directly activates PKC*γ*, a classical PKC, acting through the oxidation of the Cys residues within the C1 domain. This may occur independently of elevations in cellular DAG, the natural PKC activator [103]. In the DAG/IP3 pathway, Ca^2+^ from different intracellular sources reacts with DAG to directly activate PKC, while the phospholipase C (PLC) cleaves the phospholipid phosphatidylinositol 4,5-bisphosphate (PIP2) into IP3 and DAG. This in turn activates PKC with or without the participation of Ca^2+^, as mentioned before. IP3, in turn, triggers the opening of calcium channels to release Ca^2+^ into the cytosol. Atypical PKCs (*ζ* and *ι*) are independent of Ca^2+^ and DAG. One or several of the abovementioned mechanisms could be involved in AD brain.

Hyperglycemia-induced PKC (*β* and *δ* isoforms) have pathogenic consequences (reviewed at [104]) related to (1) activation of proinflammatory pathways through the NF-*κ*B transcription factor; (2) blood-flow abnormalities from the overexpression of eNOS and the ET-1 pathway; (3) angiogenesis and vascular permeability derived from VEGF overexpression; (4) capillary occlusion, as a consequence of collagen overproduction and excessive fibronectin due to TGF-*β*1 signaling; (5) reduced fibrinolysis related to PAI-1 activity; (6) OxS, as a consequence of ROS overproduction leaded by increased NADPH-oxidase activity.

There is no a consensus, however, about PKC activity in brain during neurodegeneration. In the AD brain, PKC may block the amyloidogenesis by phosphorylating and inactivating the glycogen-synthase kinase-3*β* (GSK-3) which stimulates the amyloidogenic pathway [105]. PKC also promotes the *α*-secretase activity to mediate cleavage of APP, favoring the nonamyloidogenic pathway [106]. As reported elsewhere, by activating the Ca^2+^-dependent *α* isoform of PKC, the antioxidant melatonin restores neurite formation, microtubule enlargement, and microfilament organization in microspikes and growth cones in cells damaged with H_2_O_2_ [107]. A PKC agonist, phorbol 12-myristate 13-acetate, causes cytoskeletal reorganization in the presence of H_2_O_2_ *in vitro*. On the contrary, by using the PKC inhibitor, bisindolylmaleimide, neurite formation, and microfilament reorganization can be blocked [108].

There are additional related to PKC as a neuroprotector; for example, the activation of PKC in brain tissues appears to prevent brain ischemia and is a target for ischemic preconditioning. Once again, the oxidation of PKC*γ* by H_2_O_2_ on the C1 domain activates the enzyme causing it to become active and possibly inhibiting gap junctions which provide a protection of cells against OxS [103]. There is an obligatory role for PKC in the induction of brain-derived neurotrophic factor (BDNF) and neurotrophin-3 (NT-3), molecular mediators of neuronal growth and homeostatic synapse activity, which are demonstrated decreased in AD brain [109]. By inhibiting* in vitro* PI3K and PKC in N2a cells, it is possible to induce GSK-3 overactivation, which further strengthens and prolongs the Alzheimer-like tau hyperphosphorylation [110].

Interestingly, A*β*, the pathological hallmark in AD, affects PKC activity. At low concentrations A*β* stimulates PKC, contributing to neurite generation. But higher concentrations of A*β* inhibit PKC activity, leading first to memory impairment and then to neuronal loss [111]. Some other protective effects attributed to PKC have been reviewed by Etcheberrigaray et al. [112] with the suggestive title “Therapeutic Effects of PKC Activators in Alzheimer's Disease Transgenic Mice.” Indeed, opposite to the idea that the activity of PKC is accompanied by a chain of deleterious effects during neurodegeneration, rather the inactivity of PKC is the real threat for the progression of AD [12].

PKC is implicated in vascular alterations, however, as observed in diabetes. In AD also A*β* deposits in vascular endothelium inhibit the activity of endothelial nitric oxide synthase (eNOS), because of a PKC-mediated phosphorylation on Ser^660^, a key step in the activation of eNOS. Indeed, using a selective inhibitor of calcium-dependent PKC investigators have rescued eNOS and NO production, allowing vasorelaxation [56]. Vascular alterations in diabetes along with cognitive impairment are the visible result of diabetes-induced brain damage. By using magnetic resonance imaging (MRI) scans, a cortical atrophy in T2D patients which resembles preclinical AD patterns has been observed [113].

It is also known that some specific PKCs (-*α*, -*δ*, -*ε*, and -*ζ*) are directly involved in multiple steps of TLR promoting neuroinflammation [114]. It is worth noting that PKC-*δ* and -*ε* do not require Ca^2+^, only DAG for their activation. Moreover, PKC-*ζ* does not require either Ca^2+^ or DAG to be activated. TLR leads the priming step to activate NF-*κ*B transcription factor which initiates the NLRP3 multiprotein complex.

The insulin-degrading enzyme (IDE) is a highly conserved zinc metallopeptidase, particularly abundant in brain; this suggests a significant association between T2D and AD. Importantly, IDE degrades insulin, IGF-1, and A*β*, and it is also a redox-regulated enzyme.

Briefly, once insulin or IGF-1 bind their corresponding receptors in brain cells, the activation of IRS induces a cascade of events leading to the neuroprotective phosphoinositide-3-kinase–protein kinase B/Akt (PI3K/PKB/Akt) pathway. PKB/Akt signaling may (1) phosphorylate and inactivate GSK-3, (2) induce CREB, and (3) activate IDE. It is widely known that under oxidative conditions, GSK-3 generates A*β* and becomes involved in tau phosphorylation, both AD landmarks [105, 115, 116].

In the context of two overlapping pathologies, with the same oxidative background as it is the case for T2D and AD, it is difficult to attribute some interference with a pathway to only one mechanism. For example, in addition to all the mentioned mechanisms, A*β* may directly interfere with the PI3K/PKB/Akt pathway by preventing the interaction between PDK and Akt. Such an interaction would allow the activation of Akt and a very complex chain of events linked to Akt [117]. A*β* oligomers, particularly A*β*-derived diffusible ligands (ADDL), may compete for the insulin receptor, transforming cells into insulin-resistant cells [118]. Thus, the insulin receptor and other cognate receptors, as well as IGF-1, are documented to be dysregulated in AD brain [119], in such a manner that they can be intrinsic defects linked to A*β* pathology.

A*β* activates NF-*κ*B-dependent neuroinflammatory pathways as well as oxidative stress. This latter response is mediated by microglia and astrocytes and intracellularly mainly by mitochondrial failure. The two metallopeptidases capable of degrading A*β* are neprilysin and IDE. The latter is directly related to insulin resistance, and it is also a redox-regulated enzyme. Thus, it is possible to speculate that the A*β*-induced oxidative stress itself might directly downregulate IDE, facilitating the accumulation of A*β*.

Five months of exposure to a high-fat diet resulting in a noninsulin dependent form of diabetes-like insulin resistance was demonstrated to cause a >2-fold elevation in amyloidogenic A*β*1–40 and A*β*1–42 peptide content in the hippocampus of 9-month-old Tg 2576 mice, relative to normoglycemic Tg 2576 mice [120]. The effect was attributed to insulin resistance which is responsible for an increased overactivity of the amyloidogenic *γ*-secretase in addition to reduced activity of IDE. The importance of IDE has been revealed in IDE^−/−^ mice, which accumulate A*β* in brain and become hyperinsulinemia and glucose intolerant [121]. There is a direct interaction of IDE with A*β* for effectively degrading it [122].

The inhibition of IDE can be an intrinsec phenomena related to AD, since the overproduction of free radicals as observed in this neurodegenerative disorder may affect directly IDE. Oxidizable thiol residues of IDE have been located to the C178, C812, and C819 amino acid residues as reported in a comprehensive mutational analysis of 13 cysteine residues within IDE [123]. This ubiquitous zinc-metalloprotease is a thiol-sensitive enzyme which can be inhibited by nitric oxide, as well as by oxidized glutathione through glutathionylation [124]. H_2_O_2_ also may interact directly with and deactivate IDE [125]. HNE, a reactive aldehyde, may form an adduct with IDE in order to deactivate it [125]. HNE is a lipid peroxidation derivative and also a powerful alkylating agent, commonly found in AD brain [21]. The diminished IDE activity, as observed in aged rats [126], is also related to long-chain free fatty acids which exhibit an inhibitory effect on this highly conserved zinc metallopeptidase, as observed* in vitro* [127]. These facts allow the speculation that OxS is a prerequisite for decreasing IDE activity, as happens in AD and T2D; this facilitates the progression toward memory impairment [128].

Finally, another oxidizable enzyme, important to both of these overlapping degenerative pathologies, T2D and AD, is the glyceraldehyde-3-phosphate dehydrogenase (GAPDH). During T2D, the overproduction of superoxide anion associated with hyperglycemia suppresses the redox-sensitive GAPDH whose primary role in glycolysis is to catalyze the conversion of glyceraldehyde-3-phosphate to 1,3-bisphosphoglycerate. GAPDH suppression is a poly (ADP-ribose) polymerase- (PARP-) mediated mechanism [129]. In this manner, the glycolytic pathway is interrupted and deviates toward the hexosamine pathway, PKC activation, and the AGE pathway. On the other hand, GAPDH has been also linked to neurodegeneration.

Apart from its classical role in glycolysis, new roles for the redox-regulatable GAPDH have been described [130]. A relevant one is in neuronal cell death triggered by oxidative stress. Its translocation to the nucleus is considered an important step in glucose-induced apoptosis, as observed in retinal Muller cells [131]. GAPDH translocates into the nucleus under a variety of stressors, particularly oxidative stress, and it is considered a sensor of nitric oxide (NO) stress [132]. GAPDH has been shown to interact with neurodegenerative disease-associated proteins, including APP [133].

It is under oxidative stress, as observed in neurodegeneration, that oxidative modifications may impart a toxic gain-of-function in GAPDH [134]. GAPDH has several oxidizable cysteines, one of which is located in the catalytic domain where it may undergo oxidation (S-nitrosylation) by NO, and binds to Siah1, an E3 ubiquitin ligase. Thereafter the complex GAPDH-Siah1 translocates to the nucleus where it causes a degradation of Siah1 substrates, which are cytotoxic. [132]. Indeed, S-nitrosylation of GAPDH and *α*-enolase, another key glycolytic and multifunctional enzyme related to neurodegeneration (its classical role is to catalize the dehydration of 2-phosphoglycerate to phosphoenolpyruvate), have been found in brains of AD and multiple sclerosis patients [134].

## 7. Concluding Remarks

Two degenerative mechanisms exist in the same organ, the brain. Both pathological processes produce excessive amounts of free radicals, leading to OxS, which in turn worsens the pathological progression for both AD and T2D. By using the same oxidative stress-dependent mechanisms, diabetes and Alzheimer may feedback on each other, accelerating the neurodegenerative process.

The neurotoxic A*β* tends to accumulate in brain in line with insulin accumulation. A*β* by itself contributes to insulin resistance in brain cells which feeds the vicious cycle between OxS and neuroinflammation [21]. It is a cause and consequence of increasing OxS. There are many hypotheses related to the causes of AD, and the cascade of A*β* is only one of them. Conversely, hyperphosphorylation of tau in AD brain responds to GSK-3, which is identified with insulin resistance in diabetes. Tau may also be phosphorylated by cyclin-dependent protein kinase 5 (cdk5), cAMP-dependent protein kinase (PKA), and stress-activated protein kinases [135]. Tau hyperphosphorylation depends heavily on oxidative stress and Ca^2+^ dysregulation, both intrinsic to AD pathogeny [21]. OxS and Ca^2+^ dysregulation, the same as neuroinflammation, are dysfunctional mechanisms related to multiple signaling pathways in addition to insulin resistance. But it is important to note that these phenomena feedback on each other, forming a pathological vicious cycle. Oxidative stress is the common factor for AD and T2D. Oxidative stress is a key factor which determines the extent and the progression of damage. T2D and AD might not necessarily be the same pathologies; they do share some insulin resistance-related mechanisms, and they are two pathologies that overlap each other in the same organ, under the same pathogenic background: oxidative stress.

## Abbreviations

5XFAD: Five times familial Alzheimer's disease mouse model of brain amyloidosis
ACC: Acetyl-CoA carboxylase
AchE: Acetylcholinesterase
AD: Alzheimer's disease
ADDL: A*β*-derived diffusible ligands
AGEs: Advanced glycation end products
APP: Amyloid precursor protein
AR: Aldose reductase
ATP: Adenosine triphosphate
A*β*: Amyloid beta
B/Akt: Protein kinase B or Akt
BDNF: Brain-derived neurotrophic factor
CaM: Calmodulin
CaMK: Calmodulin-dependent protein kinase
cAMP: Response element-binding protein
CARD: Caspase activation and recruitment domain
CCAAT motif: Cytidine-cytidine-adenosine-adenosine-thymidine
cdk5: Cyclin-dependent protein kinase 5
ChoRE: Carbohydrate response element
ChREBP: Carbohydrate response element-binding protein
COX-2: Cyclooxygenase 2, also known as prostaglandin-endoperoxide synthase 2
CREB: cAMP response element-binding protein
DAG: Diacylglycerol
DAMP: Damage associated molecular pattern molecules
ER: Endoplasmic reticulum
ERK: Extracellular signal-regulated kinase
FAS: Fatty acid synthase
G6PD: Glucose-6-phosphate dehydrogenase
GAPDH: Glyceraldehyde-3-phosphate dehydrogenase
GCL: Glutamate cysteine ligase
Grx: GlutaredoxiN
GSH: Glutathione (reduced)
GSK-3: Glycogen synthase kinase 3
GSSG: Glutathione disulfide
HNE: 4-Hydroxynonenal
IDE: Insulin-degrading enzyme
IDH: Isocitrate dehydrogenase
IGF: Insulin-like growth factor
IP3: Inositol 1,4,5-trisphosphate
IRE1*α*: Inositol-requiring protein-1 alpha
IRK: Insulin receptor kinase
IRS: Insulin receptor substrate
L-PK: L-Pyruvate kinase
MAPK: Mitogen-activated protein kinase
MDA: Malondialdehyde
MRI: Magnetic resonance imaging
NAD^+^: Nicotinamide adenine dinucleotide
NADH: Nicotinamide adenine dinucleotide (reduced form)
NADP: Nicotinamide adenine dinucleotide phosphate
NADPH: Nicotinamide adenine dinucleotide phosphate (reduced form)
NF-Y: Nuclear factor Y
NF-*κ*B: Nuclear factor-kappaB
NLRP3: Nucleotide-binding domain, leucine-rich-repeat-containing family, pyrin domain-containing 3
NOD: Nucleotide-binding oligomerization domain
NT-3: Neurotrophin-3
OxS: Oxidative stress
PARP: Poly (ADP-ribose) polymerase
PC12: Pheochromocytoma cell line 12
PDK: Pyruvate dehydrogenase kinase
PI3K: Phosphatidylinositol-3-kinase
PIP2: Phosphatidylinositol 4,5-bisphosphate
PIP3: Phosphatidylinositol (3,4,5)-trisphosphate
PKA: Protein kinase A
PKC: Protein kinase C
PLA2: Phospholipase-A2
PTEN: Protein phosphatase and tensin homolog
PTP: Protein-tyrosine phosphatase
PTP1B: Protein tyrosine phosphatase
RAGE: Receptor for AGE
ROS: Reactive oxygen species
SDH: sorbitol dehydrogenase
SR: Scavenger receptor
T2D: Type 2 diabetes mellitus
TLR: Toll-like receptor
Trx: Thioredoxin
TxNIP: Thioredoxin-interacting protein.
